# AK-Score: Accurate Protein-Ligand Binding Affinity Prediction Using an Ensemble of 3D-Convolutional Neural Networks

**DOI:** 10.3390/ijms21228424

**Published:** 2020-11-10

**Authors:** Yongbeom Kwon, Woong-Hee Shin, Junsu Ko, Juyong Lee

**Affiliations:** 1 Department of Chemistry, Kangwon National University, Gangwon-do, Chuncheon 24341, Korea; ybkwon@arontier.co; 2 Department of Chemical Science Education, Sunchon National University, Jeollanam-do, Suncheon 57922, Korea; 3 Arontier, 241 Gangnam-daero, Seocho-gu, Seoul 06735, Korea

**Keywords:** protein-ligand binding affinity, convolutional neural network, ResNext, deep learning, binding affinity prediction, docking score

## Abstract

Accurate prediction of the binding affinity of a protein-ligand complex is essential for efficient and successful rational drug design. Therefore, many binding affinity prediction methods have been developed. In recent years, since deep learning technology has become powerful, it is also implemented to predict affinity. In this work, a new neural network model that predicts the binding affinity of a protein-ligand complex structure is developed. Our model predicts the binding affinity of a complex using the ensemble of multiple independently trained networks that consist of multiple channels of 3-D convolutional neural network layers. Our model was trained using the 3772 protein-ligand complexes from the refined set of the PDBbind-2016 database and tested using the core set of 285 complexes. The benchmark results show that the Pearson correlation coefficient between the predicted binding affinities by our model and the experimental data is 0.827, which is higher than the state-of-the-art binding affinity prediction scoring functions. Additionally, our method ranks the relative binding affinities of possible multiple binders of a protein quite accurately, comparable to the other scoring functions. Last, we measured which structural information is critical for predicting binding affinity and found that the complementarity between the protein and ligand is most important.

## 1. Introduction

Predicting the binding affinity of a protein-ligand complex plays a central role in drug design and discovery. For a molecule to be a lead molecule for drug discovery, generally, it is required to bind with a target protein tightly. However, the experimental measurement of protein-ligand binding affinity is difficult and time-consuming, which is one of the major bottlenecks of the drug discovery process. If one can predict the affinity of a specific ligand to a target protein quickly and accurately, the efficiency of in silico drug discovery would be significantly improved. Thus, to accelerate the drug discovery process, many computational binding affinity prediction methods have been developed [1,2,3]. Generally, traditional methods for binding affinity prediction are classified into three categories: (1) physics-based, (2) empirical, and (3) knowledge-based methods.

The first approach, the physics-based scoring function, mainly uses theoretically rigorous binding free energy calculations based on molecular mechanics models. For this purpose, the most widely used approach is to perform free energy perturbation (FEP) calculations to estimate relative and absolute binding free energies. The strongest advantage of physics-based methods is that, with the help of state-of-the-art forcefield models, they predict the binding free energies of arbitrary small-molecule ligands to a protein accurately. Wang et al. demonstrated that a series of relative free energy calculations yield the binding free energy values of more than 200 protein-ligand complexes with an average error of about 1~2 kcal/mol [1,4,5]. It should be noted that this level of accuracy is achievable for well-behaving protein-ligand complexes. For more challenging systems, where entropic effects are large or water molecules play critical roles in determining binding affinity, the accuracy becomes worse, and obtaining converged free energy calculation results within a reasonable time frame is not guaranteed [6,7,8]. In general, rigorous free energy calculations require a significant amount of computational resources. With state-of-the-art molecular dynamics (MD) and FEP programs running on a graphics processing unit (GPU), binding free energies of only one or two ligands can be calculated within a day or two depending on the sizes of a ligand and a protein. Such computational burden hinders the extensive use of free energy calculations for high-throughput screening of drug-like molecules.

Several studies showed that MD simulations can be used for improving docking results with reasonably fast computation speed due to the improvements of GPUs and MD programs. Combined with fast theoretical methods, such as linear interaction energy [9,10], MD simulations are routinely applied to a large number of protein-ligand complexes [11,12]. Rastelli et al. used MD simulations together with the MM-GBSA and MM-PBSA approach to refine the docking pose of scores obtained with conventional ligand-docking programs [11]. They showed that refinement using MD improved the accuracy of docking poses and the enrichment of docking results. Tatum et al. combined rigid docking, MD, and the linear interaction approach to calculate relative binding affinities of multiple binding poses of EthR inhibitors [12].

Empirical scoring functions have been extensively used in many protein-ligand docking programs and virtual screening processes [13,14]. They approximate protein-ligand interactions using equations consisting of several physics-based terms, mimicking van der Waals interaction, solvation free energy, electrostatic interactions, etc. The parameters of the physics-based terms are generally fitted with experimental data to reproduce measured binding affinity values. Because of the simplicity of calculation and their close relationship with physics-based interactions, empirical scoring functions are still actively developed. The empirical scoring functions have been implemented to various docking programs: DOCK [15,16], AutoDock [17], AutoDock Vina [18], Glide [19], GOLD [20], FlexX [21], and Surflex-DOCK [22]. There are also popular scoring functions to re-score docked poses to get better results: X-Score [23], ChemScore [24], and ChemPLP [25].

Knowledge-based scoring functions are derived from protein-ligand complex structures. The basic assumption of the knowledge-based method is highly frequent atomic pairs contribute more to a binding affinity than the less frequent ones. To obtain the frequencies, distances between protein-ligand interacting atomic pairs from the 3-D structures are statistically analyzed. These data are further converted to a pseudopotential to predict a binding affinity. The advantage of knowledge-based scoring functions is the computing cost since they only require distance calculation. However, it is hard to set a reference state, an atom-randomized state, when converting the frequencies to the pseudopotential, as pointed out by Thomas and Dill [26]. Examples of knowledge-based scoring functions are DrugScore [27], IT-Score [28], SMoG [29], DFIRE [30], and PMF [31].

Recently, due to the emergence of deep-learning methods, more accurate data-driven predictions have become possible in various scientific disciplines [32,33]. For protein-ligand binding affinity prediction, many deep learning-based methods have been suggested [2,3,34]. Here, we briefly review a few examples. Ragoza et al. suggested a small network consisting of three sequential layers of a 3-D convolutional neural network (3-D-CNN) with pooling layers [35]. Similarly, Stepniewska-Dziubinska et al. developed a binding affinity prediction model consisting of three consecutive 3-D-CNN layers followed by three dense layers [36]. Jimeńez et al. developed a binding affinity prediction model, K_DEEP_, based on the SqueezeNet architecture [37], which was originally designed for image classification [38]. The K_DEEP_ model consists of multiple 3-D-CNN with about 1.3 million parameters. Zhang et al. developed the DeepBindRG model [39], which uses the 2-D representation of a protein–ligand interface and the ResNet architecture [40]. Similarly, Zheng et al. also converted a protein-ligand binding structure into a 2-D tensor with a single channel and processed it through three 2-D-CNN layers and four dense layers [41].

In this paper, we present a new protein-ligand binding affinity prediction model, which estimates the binding affinity of a complex based on a given 3-D structure. Our model was inspired by the ResNext architecture [42], which uses an ensemble of CNN filters and showed an improved image classification accuracy compared to its precedents. In addition to using a novel network architecture compared to previous models, we demonstrate that an ensemble-based approach, using an average of multiple predictors instead of a single predictor, significantly improves prediction quality. The advantage of an ensemble approach is that it does not require further modification of network architectures and can be readily applicable to most existing models. The benchmark results using the Comparative Assessment of Scoring Functions 2016 (CASF-2016) dataset [43] show that the performance of our model is comparable to the best existing scoring functions. We also analyzed the relative feature importance to gain insights on which physical properties are most essential in determining binding affinity.

## 2. Results and Discussion

### 2.1. Binding Affinity Prediction Accuracy

**Figure 1:**
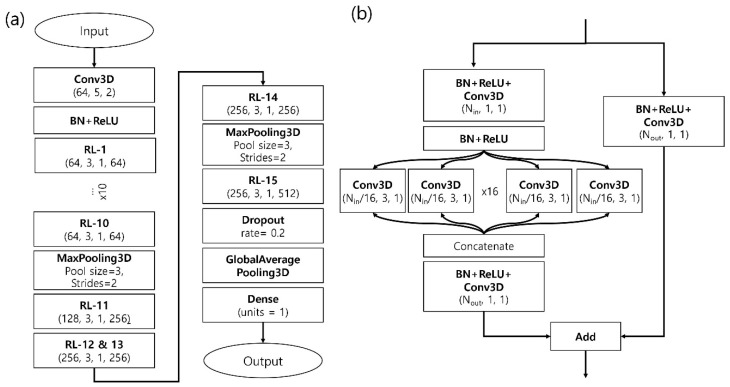
(**a**) The overall architecture of the network. The complete network mainly consists of 15 stacked layers of an ensemble-based residual layer (RL) block. The parentheses in the RL blocks denote (number of input channels, kernel size, stride, number of output channels). The numbers inside parentheses in the Conv3D blocks represent (number of channels, kernel size, stride). (**b**) The structure of each residual block is illustrated. A residual block consists of three stacks of convolutional layers combined with batch normalization and ReLU activation layers and a residual sum. In the middle of the block, each subset of channels is distributed to 16 separated 3-D-convolutional layers and processed in a parallel way. After being processed by the parallel convolutional layers, the output tensors are concatenated by the channel axis. Then, the agglomerated tensor is processed with a 3-D-convolutional layer with a kernel size of one to adjust the number of channels and added with a shortcut tensor, which is similar to an identical tensor of the original residual network (shown as the right path). Abbreviations are defined as following: Conv3D: 3-D convolutional neural network layer, BN: Batch Normalization layer, RL: Residual Layer.

We performed two different types of scoring schemes based on AK-score architecture: AK-score-single, and AK-score-ensemble. AK-score-single uses a single prediction network as shown in Figure 1. AK-score-ensemble uses an average of 20 independently trained networks as the final prediction value. The mean absolute error (MAE) and root mean squared error (RMSE) between predicted and experimental values of various models trained with different learning rates are listed in Table 1. For comparison, the results of the other 3-D-CNN-based deep-learning model, the K_DEEP_ model, implemented by our group are also provided.

The benchmark results show that the AK-score-ensemble model yields the most accurate prediction results. Among the tested models, AK-score-ensemble has the lowest accuracy metric values with an MAE of 1.01 kcal/mol and an RMSE of 1.29 kcal/mol. Compared with a single model, the average errors of the ensemble model are lower by about 0.1 kcal/mol. Besides, when compared with the K_DEEP_ model, AK-score-ensemble has a lower average error by 0.2 kcal/mol. Our results also show that choosing the best learning rate improves MAE by about 0.05 kcal/mol and RMSE by 0.10 kcal/mol.

We also assessed the performance of our models based on three criteria used in the CASF-2016 dataset, scoring, ranking, and docking power (Table 2). In all three criteria, the AK-score-ensemble model shows the best performance. Scoring power is indicated by the Pearson correlation coefficient between predicted and experimental values. Overall, AK-score models result in higher correlation values than the K_DEEP_ model. Among all the tested models, only AK-score-ensemble yields a correlation coefficient value higher than 0.8. In terms of ranking power, the AK-score models outperform the K_DEEP_ model on average. Among them, the AK-score-ensemble model results in the highest rank correlation coefficients. For docking power, the difference in prediction performance of the K_DEEP_ and the AK-score-single model is not as prominent as in the other criteria. However, the prediction results of the AK-score-ensemble model are better than those of the K_DEEP_ model.

### 2.2. An Ensemble of Networks Improves the Quality of Prediction

**Figure 2:**
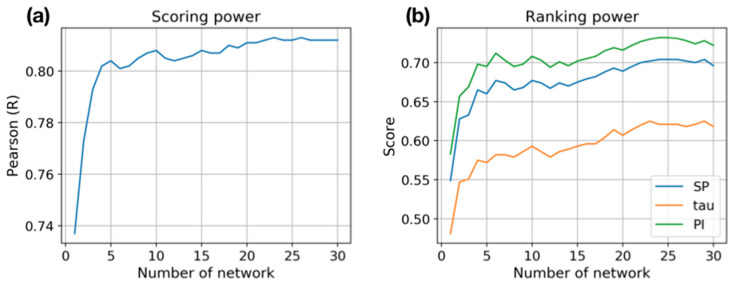
The change of prediction quality by the size of the ensemble of networks. (**a**) Scoring power is measured by the Pearson correlation coefficient between the experimental and predicted binding affinities. (**b**) Ranking power is measured by three rank correlation coefficients, Spearman (SP), Kendall tau (tau), and predictive Index (PI).

We investigated the change of prediction quality by the number of networks. The results show that overall the prediction accuracy increases as more networks are used. In both metrics, scoring, and ranking power, the accuracy rapidly increases from a single network to five networks. In terms of the scoring power, the Pearson R correlation coefficient between experiments and predictions increases until the number of networks reaches 25 (Figure 2a). When a single network is used, the correlation coefficient is less than 0.74. However, when the average of the five networks is used, the value becomes higher than 0.80. After 10 networks, the improvement becomes modest but is kept until 25 networks are used. Similarly, the ranking power keeps improving until 25 networks are used. All three ranking measures (Spearman correlation coefficient (SP), Kendall tau, and Predictive index (PI)) are improved consistently when the ensemble average of networks is employed (Figure 2b). These results clearly show that using the ensemble of prediction networks significantly improves prediction quality, which is a simple and straight-forward way to improve prediction accuracy without further exploration of various network architectures.

### 2.3. Comparison with Other Scoring Functions

**Figure 3:**
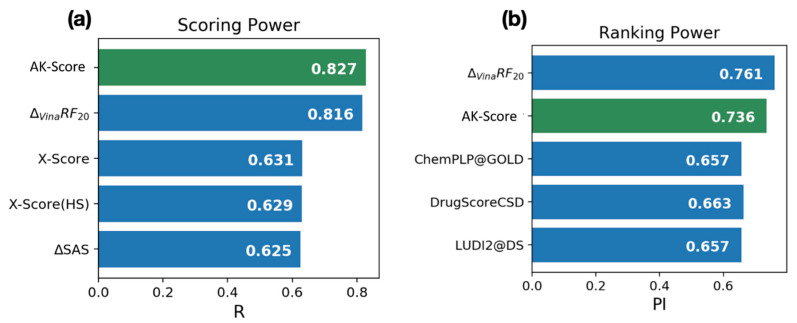
Benchmark results of AK-score with existing protein-ligand binding affinity scoring functions; (**a**) Scoring power (**b**) Ranking power. In each panel, AK-score is shown in green, and the other top 4 scoring functions from Su et al. [43] are shown in blue.

The benchmarking result of the AK-score shows that its prediction accuracy is comparable with the best existing scoring functions based on the CASF-2016 dataset. The CASF-2016 dataset provides the pre-calculated prediction results of known scoring functions, which allows a fair comparison of our model with existing scoring functions using the same test set. The comparison results of scoring and ranking with the top-scoring functions of each category are shown in Figure 3. Overall, the result of the AK-score-ensemble model with 30 independently trained networks (green) shows the best correlation coefficient, 0.827, among the tested scoring functions (blue) in the scoring power category (Figure 3a). The correlation coefficient is a little higher than the best value obtained with Δvina-RF20 (0.816), which employs a random forest as a predictor, and much higher than the traditional scoring functions, X-Score (0.631) and AutoDock-Vina (0.604, not shown in Figure 3a). Additionally, the ranking power of the AK-score (0.736), measured by the PI, is slightly worse than the best value by Δvina-RF20, 0.761, which corresponds to the second place among the tested scoring functions (Figure 3b).

**Figure 4:**
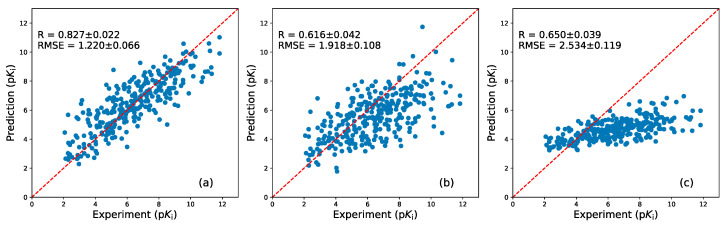
The scatter plots of the experimental binding affinities and prediction results obtained with (**a**) AK-score-ensemble, (**b**) Autodock-Vina, and (**c**) X-score are depicted. The predicted binding affinities in kcal/mol were converted to p*K_i_* to compare with the experimental binding affinities. The Pearson correlation coefficient and root-mean-squared-error (RMSE) value of the predictions and their uncertainties are estimated using the bootstrapping analysis.

Interestingly, AK-Score correlates well with experimental binding affinity values compared to all tested complexes while the Autodock-Vina and X-score, which are widely used scoring functions for the docking program, show biases clearly (Figure 4). The two scoring functions significantly underestimate absolute binding affinities, which is indicated by a small slope coefficient of a regression line (Figure 4b,c). The correlation coefficient of AK-score predictions with the experiment is 0.827 while those of Autodock vina and X-score are 0.616 and 0.650, respectively. The RMSE of AK-score predictions is 1.22 p*K_i_* unit, while those of Autodock vina and X-score are 2.62 p*K_i_* unit and 3.47 p*K_i_* unit. The statistical significance of the differences between the correlation coefficients of multiple predictions are investigated using bootstrapping samplings and t-tests; two-thirds of test set samples are randomly sampled with a replacement for 1000 times and their metrics are calculated. The average and the standard deviation of the correlation coefficients and the RMSE values of the bootstrapping sets are calculated and their t-values are calculated. The t-value between the correlation coefficients of AK-score and Autodock vina is 136.8. Additionally, the t-value between the AK-score and the X-score results is 120.8. These t-values correspond to *p*-values of virtually zero, demonstrating that the quality of AK-score regression is significantly better than those of the other scoring functions. In summary, AK-score-ensemble outperforms the widely used empirical scoring functions in absolute binding affinity prediction.

### 2.4. Assessment with an Additional Dataset

To assess the transferability of AK-score, we predicted the binding affinities of protein-ligand complexes that were not included in the training and test sets. The protein-ligand complexes that are newly included in the PDBBind-2018 after the release of PDBBind-2016 were used for the additional test. A total of 534 complexes were used as the additional test set and the list of the complexes is presented in the Supplementary Materials.

**Figure 5:**
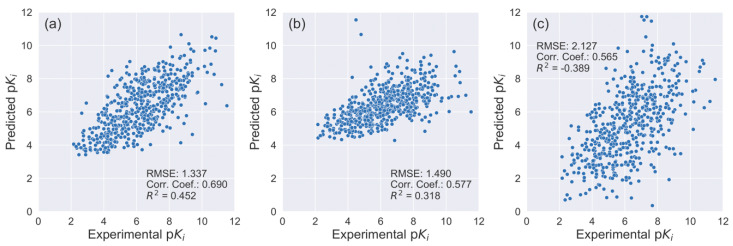
Binding affinity prediction results of protein-ligand complexes that are included in the PDBBind-2018 database but not in the PDBBind-2016: (**a**) AK-score, (**b**) X-Score, and (**c**) ChemPLP. The X-axis corresponds to experimental p*K_i_* values and the Y-axis to predicted values.

The AK-score prediction results show the RMSE of the predicted values was 1.34 p*K_i_* unit, which is comparable to the test set result. The correlation coefficient between the experimental and predicted values was 0.69 and the coefficient of determination, R^2^, was 0.45, which is slightly lower than those of the test set results. These results suggest that the performance of AK-score is transferrable to other protein-ligand complex systems. The correlation coefficient is higher than X-Score (0.58) and ChemPLP (0.56), which is consistent with the CASF-2016 benchmark result (Figure 5).

### 2.5. Identifying Hot Spots for Binding Affinity Determination Using Grad-CAM

The gradient-weighted class activation mapping (Grad-CAM) is a widely used method to interpret which features are responsible for determining outputs [44]. This approach detects the sum of the magnitude of gradients that activated the nodes of the last layer. We utilized the Grad-CAM method to identify a subset of atoms of protein-ligand binding sites that play important roles in determining binding affinity values. To achieve this, we slightly modified the architecture of the AK-score network by replacing the last single neuron with a fully connected layer with 20 nodes, which converts our regression model to a classification model. We aggregated the weights of gradients to determine important regions for protein-ligand binding.

**Figure 6:**
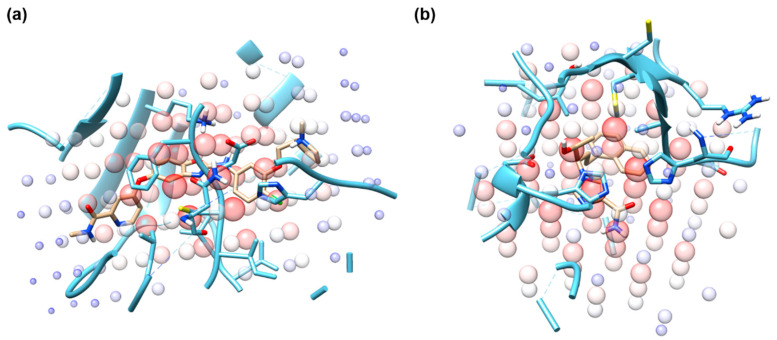
Grad-CAM analysis examples of protein-ligand complexes whose PDB IDs are (**a**) 2QU6 and (**b**) 2E1W. The size and color of transparent spheres indicate the magnitude of the sum of gradients. Receptor and ligand are colored in blue and gold, respectively.

The example cases of Grad-CAM analysis of the protein-ligand complexes with PDB ID of 1BCU and 2E1W are illustrated in Figure 6. Figure 6a is the structure of vascular endothelial growth factor receptor 2 complexed with a benzoxazole inhibitor (PDB ID: 2QU6). In the figure, Grad-CAM illustrates important regions by the size and the color of circles. The size of the circle is proportional to the magnitude of the sum of gradients and as it becomes larger, the color of the circle becomes red from blue. In this example, the most important region that determines that binding affinity of the ligand is clustered around the core aromatic fused ring. This suggests that the interactions between the protein pocket and the aromatic ring play an important role in determining the binding affinity. Figure 6b (PDB ID: 2E1W) shows an important region of adenosine deaminase and its potent inhibitor. In the example, the most important structural motif was identified to be the naphthalene ring included in the ligand and the hydroxyl group of the ligand. These examples show that combining CNN-based prediction networks with the Grad-CAM analysis may help identify critical residues in determining the binding affinity of a complex. The heatmaps of the Grad-CAM analysis of all complexes included in PDBbind-2016 are provided as Supplementary Materials.

### 2.6. Assessment of Feature Importance via Ablation Test

To obtain chemical and biological insights from the trained networks, it is necessary to identify which atomic features play important roles in determining the binding affinity of a protein-ligand complex. To achieve this goal, we performed additional experiments that perform predictions by (1) making the values of a specific channel as zero, or (2) randomly shuffling the values of a channel, which is called the ablation test [45]. The values of other channels are kept while the values of a specific channel are zeroed or shuffled. The rationale of the second experiment is based on the conjecture that making all values of a channel zero may be too drastic a loss of information and conserving the average and variance of values of a channel may be important for making reasonable predictions. After zeroing or shuffling a specific channel, we measured ΔMAE from the result of the original data. Large ΔMAE means the error increases when specific channel values are zeroed or shuffled. Thus, we can get the importance of a channel by measuring by ΔMAE from shuffling and zeroing experiments.

**Figure 7:**
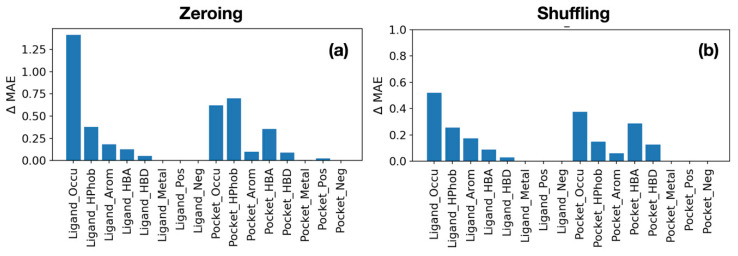
Feature importance calculation results measured by the loss of prediction accuracy, the increase of mean absolute error of predictions in kcal/mol (Y-axis). (**a**) A channel corresponding to a feature (X-axis) is filled with zero. (**b**) The values of a channel corresponding to a feature are randomly shuffled.

Overall, from both experiments, the excluded volumes of a ligand and a binding site are identified to be the most important features in determining the binding affinity of a protein-ligand complex (Figure 7). In other words, the shape complementarity between a binding site and a ligand is most important in determining the binding affinity of a complex. When excluded volume information of a ligand is missing, the average binding affinity prediction accuracy deteriorates by 1.4 kcal/mol (Figure 7a). Following the excluded volume information, the hydrophobic atom information of a ligand and a binding site is identified to be the second important factor. For a binding site, the hydrogen acceptor atoms of a binding site play the third important role. Interestingly, for ligands, aromatic atoms play the third important role. In the shuffling experiment, the overall trend is similar to that of the zeroing experiment, but the average decrease in prediction accuracy is smaller (Figure 7b). The most prominent difference is that, for a binding site, the relative importance of the hydrogen bond acceptor atoms becomes larger than that of hydrophobic atoms.

## 3. Methods

### 3.1. Data Preparation

The protein-ligand binding affinity data for training and testing the network was adopted from the PDBBind-2016 database [46]. The database is composed of protein-ligand complex structures in which their experimental binding affinities are known. All PDB structures in the database were further filtered to obtain a refined set. Conditions to get the refined set are: (1) The resolution of crystal structures are better than or equal to 2.5 Å, (2) the ligand binds to the receptor noncovalently, (3) the stoichiometry between protein and ligand in crystal structure should be 1:1, (4) the experimentally determined binding affinity is K_i_ or K_d_, and (5) the ligand should be only composed of common organic elements, i.e., C, N, O, P, S, F, Cl, Br, and I [46]. The set is composed of 4057 protein-ligand complexes. The receptors in the refined set were further clustered with 90% of sequence similarity. If the members of a cluster are larger than five, and the difference between the highest binding affinity and the lowest binding affinity is larger than 100, then the cluster was selected as a member of the core set [46]. In each cluster, five representative PDBs are selected: two complexes with the maximum and the minimum binding affinities in the cluster and the other three have a binding affinity that differs by at least one-fold (i.e., log2). The core set, composed of 285 protein-ligand complexes, was used as a test set. The remaining 3772 complexes (refined set minus core set) were used as a training set.

### 3.2. Convolutional Neural Network

To utilize the power of the convolutional neural networks, the structures of protein-ligand complexes were represented as three-dimensional (3-D) grids. We voxelized a binding pocket and a ligand following Jimeńez et al. [37]. For each protein-ligand complex, the center of mass of the bound ligand was set to the origin and the neighboring atomic environment was embedded into a 3-D grid whose edge length was 30 Å. Along the X, Y, and Z-axes, 30 grid boxes were generated with a spacing of 1.0 Å. To capture the pattern of protein–ligand interactions, for each grid box, atomic density was calculated using the following density function (Equation (1)):

(1) $$n(r)=1-\exp\left[-{\left(\frac{r_{VDW}}{r}\right)}^{12}\right],$$

where *r_VDW_* is the van der Waals radius of an atom and r is a distance between an atom and the center of a 1 Å ⋅ 1 Å ⋅ 1 Å. *n*(*r*) ranges from 0 (no contribution by the atom) to 1 (fully occupied by the atom). At each center of the grid box, the contributions from all atoms were summed up.

Atoms were classified into 8 classes, and they were represented as the different channels of the input data. We treated atoms from proteins and ligands separately, which leads to 16 real-valued channels representing the aggregated number density of each protein-ligand complex. The atoms were labeled the same as Jimeńez et al. [37]. Most of the atom types were determined after assigning AutoDock4 atom types [17], except for positive and negative atom types. They were assigned by following the sign of the Gasteiger charge of an atom. Gasteiger atomic partial charges were calculated based on the atom and bond types, equalizing the electronegativity of orbitals [47]. The description of the atom types used in this study is listed in Table 3.

To reduce the orientation dependency of a complex structure, we augmented the number of data by rotating a grid with all 24 possible rotational operations.

### 3.3. Network Architecture

The main component of our network is an ensemble-based residual network, which was used in the ResNext model for image recognition. Compared to other deep learning models, ResNext has a simple architecture but shows better performance in the image recognition benchmark [42]. The overall structure of the network is illustrated in Figure 1a and the structure of each residual block is shown in Figure 1b. At each residual block, each channel is distributed to multiple convolutional layers and processed in a parallel way. The number of parallel residual networks is also called cardinality. In this study, we used 16 3-D-convolutional layers (Conv3D) layers for each residual block, which corresponds to a cardinality of 16. We called this network architecture AK-score (Arontier-Kangwon docking scoring function).

The complete network mainly consists of 15 stacked layers of an ensemble-based residual layer (RL) block (Figure 1). A single initial input tensor has information of 16 atom-types (channels) and a 30 Å ⋅ 30 Å ⋅ 30 Å cubic, corresponding to a tensor with a shape of (16, 30, 30, 30) in the channel-first format. First, an initial tensor is processed with a 3-D-convolutional layer with 64 output channels, a kernel size of 5, and a stride of 2, which converts the shape of the output tensor into (64, 15, 15, 15). After the convolutional layer, the batch normalization and the activation layers follow. The resulting tensor is put into a series of 10 residual blocks.

A single RL layer consists of three stacks of convolutional layers combined with the batch normalization and the rectified linear unit (ReLU) activation function layer and a residual addition (Figure 1b). First, an input tensor goes through the BN and ReLU layers. In the middle of the block, each subset of channels of a tensor is distributed to 16 independent Conv3D layers and processed in a parallel way, which corresponds to a cardinality of 16. For example, if the number of input channels is 64, such as RL1 to 10, the input tensor is separated into 16 tensors with only four channels. After being processed by the parallel convolutional layers, the output tensors are concatenated by the channel axis. Then, the agglomerated tensor is processed with a Conv3D layer with a kernel size of one to adjust the number of channels and added with a shortcut tensor, which corresponds to an identical tensor of the original residual network. The total number of parameters of the network is 1,294,925. Among them, the number of trainable and non-trainable parameters is 1,293,447 and 1478, respectively.

The ReLU activation function was used for all activation layers of the network. All weight parameters were initialized with the He_normal initialization scheme. The model loss was calculated with the MAE between the experimental and predicted binding affinities in a kcal/mol unit. For parameter optimization, the Adam optimizer was used with the following parameters: beta-1 = 0.99 and beta-2 = 0.999. The model was trained with multiple learning rates to test the effect of the learning rate on the final prediction quality. Learning rates of 0.0001, 0.0005, 0.0007, and 0.0010 were tested. While training the network, the whole dataset was randomly permuted to avoid possible biases.

### 3.4. Ensemble Prediction

To enhance prediction accuracy, we employed an ensemble prediction scheme, obtaining the final prediction value from the average of multiple independently trained models. In many machine-learning tasks, the parameters of each prediction model are optimized from initial random values. When the number of parameters is large, the final parameter set does not converge well in general. To reduce possible such biases, we trained multiple networks independently and assessed whether the average of multiple predictions yielded better predictions. We called this ensemble-based model AK-score-ensemble. For comparison, a model based on a single network is called AK-score-single in this paper.

### 3.5. Performance Assessment

To assess the performance of our model, we compared our model with the previously suggested 3-D-CNN based binding affinity prediction model. We implemented the K_DEEP_ model, which is based on SqueezeNet architecture, which was used for image classification [37]. The model was trained with the same parameters reported in the reference. All models reported in this study were implemented in Keras-2.2.4 with Tensorflow-1.13.1 backend (Google, Mountain View, CA, USA) [48].

As a benchmark set, we used the CASF-2016 benchmark dataset. The benchmark set has been used as a common ground for a comparison of various scoring functions [43,49,50]. Wang and his colleagues started comparing various scoring functions, called CASF, from 2007. Binding pose prediction of a docking program depends on not only a scoring function but also a sampling algorithm it uses. Thus, it is hard to evaluate the power of scoring function purely. The aim of the series of CASF benchmarks is evaluating a pure performance of scoring functions by decoupling sampling and scoring.

The main purposes of scoring functions are (1) predicting near-native binding pose, (2) estimating the binding affinity of a given pose, and (3) ranking compounds based on predicted binding affinities. Thus, the CASF benchmark evaluates the accuracy of protein-ligand docking prediction in three categories: scoring (correlation between predicted binding affinity and experimentally determined binding affinity), ranking (correlation of ranked compounds by predicted affinities and experimental values), and docking (ability to picking up native pose among decoys). The benchmark categories and their metrics are summarized in Table 4.

The first category is scoring. The aim of this category is how the scoring function correlates well with the experimentally determined binding affinity. The scoring function predicts the binding affinities of 285 crystal structures and the predicted values are compared with the experimentally determined binding affinities. The metric used in the category is Pearson correlation coefficient R (Equation (2)):

(2) $$R=\frac{\sum_{i=1}^{n}\left(x_{i}-\bar{x}\right)\left(y_{i}-\bar{y}\right)}{\sqrt{\sum_{i=1}^{n}{\left(x_{i}-\bar{x}\right)}^{2}}\sqrt{\sum_{i=1}^{n}{\left(y_{i}-\bar{y}\right)}^{2}}},$$

where *x_i_* and *y_i_* are the predicted and experimentally determined binding affinities of complex *i*. $\bar{x}$ and $\bar{y}$ are the average values of the predicted and experimental binding affinities.

The second category is ranking. Apart from the correlation, it focuses on how accurately a scoring function ranks a set of protein-ligand complexes by the predicted values when a set of protein-ligand complex structures are given. In each receptor cluster of the core set, there are five crystal structures. Thus, the ranking problem is reduced to how the scoring function correctly orders the five complexes.

To assess the ranking power, Spearman’s correlation coefficient, Kendall tau, and predictive index (PI) values are used. These metrics are measurements of the rank correlation between compounds ranked by experimentally determined affinities and by predicted affinities. All the three metrics range from −1 (totally reversed order) to 1 (perfect ranking). Spearman’s correlation coefficient *ρ* is calculated as below (Equation (3)):

(3) $$\rho =\frac{\sum_{i=1}^{n}\left\{\left[R\left(x_{i}\right)-\bar{R(x)}\right]\times \left[R\left(y_{i}\right)-\bar{R(y)}\right]\right\}}{\sqrt{\left\{\sum_{i=1}^{n}{\left[\left[R\left(x_{i}\right)-\bar{R(x)}\right]\right]}^{2}\right\}\times \left\{\sum_{i=1}^{n}{\left[\left[R\left(y_{i}\right)-\bar{R(y)}\right]\right]}^{2}\right\}}},$$

where *R*(*x_i_*) and *R*(*y_i_*) are the ranks of complex *i* by its predicted binding affinity and experimental binding affinity, respectively. $\bar{R(x)}$ and $\bar{R(y)}$ are the mean ranks, and *n* is the number of complexes.

The Spearman’s correlation coefficient is calculated based on deviations of ranking, while the Kendall tau coefficient is proportional to a difference between concordant pairs and discordant pairs. Two variable pairs (x_1_, y_1_) and (x_2_, y_2_) are called concordant if x_1_ > x_2_ and y_1_ > y_2_ (or x_1_ < x_2_ and y_1_ < y_2_). Otherwise, the pairs are called discordant. The coefficient is computed as Equation (4):

(4) $$\tau =\frac{N_{concord}-N_{discord}}{\sqrt{\left(N_{concord}+N_{discord}+T\right)\left(N_{concord}+N_{discord}+U\right)}}.$$

where *N_concord_* and *N_discord_* are the number of concordant and discordant pairs, respectively. *T* is the number of ties in predicted binding affinity, and *U* is the number of ties in experimental binding affinity.

*PI* focuses on the ranking ligands correctly with high priority if the ligands have significantly different experimental binding affinity [51]. The index is computed by two components, $\omega_{ij}$ and $S_{ij}$ (Equation (5)):

(5) $$PI=\frac{\sum_{j>i}^{n}\sum_{i}^{n}\omega_{ij}S_{ij}}{\sum_{j>i}^{n}\sum_{i}^{n}\omega_{ij}},$$

where $S_{ij}$ is 1 if the predicted binding affinities and experimental binding affinities of two complexes are concordant pairs. If they are discordant pairs, then $S_{ij}$ is set to 0. $\omega_{ij}$ equals to the experimental binding affinity difference between two complexes, so it acts as a weight for complex pairs.

The last category is docking. This benchmark tests whether a scoring function can find the native ligand binding pose when it is mixed with 100 decoy ligand conformations. This category is assessed by counting the number of cases that a scoring function ranks the native pose within the top 1, top 2, and top 3, among the given ligand conformations.

Among the series of CASF benchmark datasets, we used CASF-2016, the latest one, to evaluate AK-score performance. The set is composed of 285 protein-ligand complexes, the same as the core set of PDBBind-2016. We compared the performance first with K_DEEP_ and later with the 25 scoring functions assessed in Su et al. [43].

## 4. Conclusions

We developed a new binding affinity prediction model, AK-score, by combining a multi-branched deep-learning network architecture, ResNext, and an ensemble predictor approach. We first compared the AK-score-ensemble model with AK-score-single, a single predictor model with ResNext, and K_DEEP_, which employs ResNet as a deep learning architecture. The AK-score-ensemble model showed lower MAE and RMSE than the others. Then, we also tested our predictor on the CASF-2016 dataset. Our model predicts the binding affinity of a protein-ligand complex with a high accuracy, which is comparable to the best existing scoring functions in terms of scoring and ranking power. In the scoring category, our predictor showed the best Pearson correlation coefficient among the tested 26 scoring functions, and in the ranking category, AK-score ranked second when the ranking power was measured by PI. Additionally, we showed that the deep learning model could be transferred to a new complex, by testing it with PDB structures included in PDBBind-2018, but not members of PDBBind-2016. Our results suggest that an ensemble-based approach, using the average of multiple independently trained models, is a straightforward but powerful approach. A similar approach may apply to existing machine-learning-based models. Additionally, our study gives an insight into the relative importance of atoms based on their chemical properties. The feature importance tests show that, for a ligand, the excluded volume of atoms, the spatial distribution of hydrophobic and aromatic atoms, is critical in determining binding affinity. For a protein, the excluded volume of atoms, and the distribution of hydrophobic atoms and hydrogen bond acceptors are identified to be important factors. We believe that our results provide useful guidelines for the development of next-generation deep-learning-based protein-ligand scoring functions.

## Figures and Tables

**Table 1:** Assessment of prediction accuracy of ResNext-ensemble, ResNext, and K_DEEP_ using the PDBbind-2016 dataset and mean absolute error and root mean square error metrics.

Model	Learning Rate	MAE (kcal/mol)	RMSE (kcal/mol)
K_DEEP_	0.0001	1.131	1.462
	0.0005	1.200	1.519
	0.0006	1.164	1.534
	0.0010	1.219	1.536
AK-score-single	0.0001	1.159	1.511
	0.0005	1.101	1.415
	0.0007	1.130	1.425
	0.0010	1.110	1.406
AK-score-ensemble	0.0007	**1.014**	**1.293**

The lowest values are shown in bold.

**Table 2:** A comparison of prediction accuracy with the CASF-2016 dataset.

Model		Scoring	Ranking			Docking		
	learning rate	Pearson (R)	Spearman (SP)	Kendall (tau)	Predictive Index (PI)	Top 1 (%)	Top 2 (%)	Top 3 (%)
K_DEEP_	0.0001	0.738	0.539	0.435	0.559	24.8	38.5	52.2
	0.0005	0.709	0.486	0.389	0.535	29.1	39.9	49.6
	0.0006	0.701	0.528	0.439	0.558	29.1	39.9	49.6
	0.0010	0.715	0.479	0.400	0.492	24.8	36.3	44.6
AK-score-single	0.0001	0.719	0.572	0.456	0.600	34.9	48.6	56.1
	0.0005	0.755	0.596	0.512	0.616	29.9	43.2	54.0
	0.0007	0.759	0.616	0.526	0.640	31.3	47.1	57.9
	0.0010	0.760	0.598	0.505	0.627	26.3	43.9	54.0
AK-score-ensemble	0.0007	**0.812**	**0.670**	**0.589**	**0.698**	**36.0**	**51.4**	**59.7**

The highest values are shown in bold.

**Table 3:** Atom types used to classify atoms forming protein-ligand binding sites.

Atom Type	Definition
Hydrophobic	Aliphatic or aromatic C
Aromatic	Aromatic C
Hydrogen bond donor	Hydrogen bonded to N, O, or S
Hydrogen bond acceptor	N, O, and S with lone electron pairs
Positive	Ionizable Gasteiger positive charge
Negative	Ionizable Gasteiger negative charge
Metallic	Mg, Zn, Mn, Ca, or Fe
Excluded Volume	All atom-types

**Table 4:** Summary of CASF-2016 benchmark categories.

Category	Aim of a Category	Metric
**Scoring**	How well the scoring function correlates with the experimental binding affinities?	Pearson correlation coefficient
**Ranking**	How relative order of binding affinities is correctly predicted?	Spearman correlation coefficient Kendall tau Predictive index
**Docking**	Can the scoring function find the native ligand binding pose?	Top 1 (%), Top 2 (%), Top 3(%)

## Supplementary Materials

Supplementary materials can be found at https://www.mdpi.com/1422-0067/21/22/8424/s1. Supplement 1: A list of PDB IDs in PDBBind-2018, not included in PDBBind-2016; Supplement 2: Grad-CAM results of PDBBind-2016 refined set.

## Abbreviations

FEP	Free energy perturbation
MD	Molecular dynamics
GPU	Graphics processing unit
CASF	Comparative assessment of scoring functions
3-D-CNN	3D convolutional neural network
Conv3D	3D convolutional neural network layer
AK-score	Arontier-Kangwon docking scoring function
BN	Batch normalization
RL	Residual layer
ReLU	Rectified linear unit
MAE	Mean absolute error
PI	Predictive index
RMSE	Root mean squared error
