# Supplementary material for: AK-Score: Accurate Protein-Ligand Binding Affinity Prediction Using an Ensemble of 3D-Convolutional Neural Networks
Source: Int J Mol Sci. 2020 Nov 10;21(22):8424. doi: 10.3390/ijms21228424 (PMC7697539; doi:10.3390/ijms21228424)
Supplement: Supplementary file 1 [file ijms-21-08424-s001.zip › Supplementary_material_1.docx]

**Supplementary material 1.** List of 534 complexes

4d7b, 4mq6, 4muf, 4mul, 4r06, 4uc5, 4uj1, 4uj2, 4uja, 4ujb, 4und, 4xk9, 4xo8, 4xoc, 4xoe, 4y3j, 4y3y, 4y4j, 4ybk, 4yhm, 4yho, 4yk0, 4z07, 4zgk, 4zt8, 4zv1, 4zv2, 5a6k, 5a7y, 5aa9, 5aan, 5ant, 5anu, 5anv, 5aqz, 5ave, 5avf, 5ayt, 5b2d, 5b5f, 5b5g, 5btv, 5bw4, 5c2o, 5chk, 5cj6, 5cks, 5cp5, 5cqt, 5cs6, 5cso, 5csp, 5cst, 5cu4, 5cy9, 5d0c, 5d0r, 5d24, 5d25, 5d26, 5d3h, 5d3j, 5d3l, 5d3n, 5d3p, 5d3t, 5d3x, 5d45, 5d47, 5d48, 5d6j, 5dbm, 5dex, 5dey, 5dfp, 5dhu, 5dkn, 5dlx, 5dnu, 5dqc, 5dqf, 5dus, 5duw, 5dw2, 5dx4, 5dxt, 5dyo, 5e2l, 5e6o, 5e73, 5e74, 5e7n, 5e89, 5ect, 5edb, 5edc, 5edd, 5edl, 5egu, 5ehq, 5ehr, 5ei3, 5eis, 5en3, 5epl, 5epn, 5eq1, 5eqe, 5eqp, 5eqy, 5er4, 5etb, 5etj, 5eu1, 5evz, 5ewk, 5ewy, 5exl, 5exm, 5exn, 5exw, 5ey0, 5ey4, 5eyr, 5f08, 5f0f, 5f1h, 5f1r, 5f1v, 5f1x, 5f25, 5f2p, 5f2r, 5f2u, 5f5z, 5f60, 5f61, 5f62, 5f63, 5f74, 5f8y, 5f9b, 5fbi, 5fck, 5fcz, 5fe6, 5fe7, 5fe9, 5fh7, 5fh8, 5fhm, 5fhn, 5fho, 5fnc, 5fnd, 5fnf, 5fnr, 5fns, 5fnt, 5fnu, 5fog, 5fol, 5fot, 5fou, 5fov, 5fox, 5fpk, 5fs5, 5fsn, 5fso, 5fsx, 5fsy, 5ftg, 5fto, 5fut, 5fwr, 5fyx, 5g1z, 5g2g, 5g46, 5g4m, 5g4n, 5g4o, 5g5f, 5g5z, 5g60, 5g61, 5gmh, 5gof, 5gs9, 5gsa, 5h1t, 5h1u, 5h1v, 5h5f, 5h8e, 5h8g, 5h9r, 5ha1, 5hbn, 5hbs, 5hct, 5hcv, 5hcy, 5hjq, 5hrv, 5hrw, 5hrx, 5htl, 5htz, 5hu9, 5hvs, 5hvt, 5hwv, 5hz5, 5hz6, 5hz8, 5hz9, 5i1q, 5i29, 5i2e, 5i2f, 5i3v, 5i3w, 5i3x, 5i3y, 5i7x, 5i7y, 5i80, 5i88, 5i8g, 5i9x, 5i9y, 5i9z, 5ia0, 5ia1, 5ia2, 5ia3, 5ia4, 5ia5, 5ie1, 5igm, 5ih9, 5ihh, 5ii2, 5ikb, 5ime, 5ioz, 5ipc, 5ipj, 5irr, 5isz, 5ito, 5itp, 5izf, 5izj, 5j0d, 5j1r, 5j3l, 5j41, 5j6a, 5j7q, 5j7w, 5ja0, 5jfp, 5jfu, 5jg1, 5jhb, 5ji8, 5jop, 5jox, 5jq5, 5jsg, 5jsj, 5jsq, 5jxq, 5jy3, 5jzi, 5k03, 5k0h, 5k1d, 5k1f, 5k8s, 5k9w, 5ka1, 5ka7, 5ka9, 5kab, 5kad, 5kat, 5kax, 5kbe, 5kby, 5kcb, 5kej, 5khm, 5kly, 5km9, 5kma, 5ko1, 5kqx, 5kqy, 5kr0, 5kr1, 5kr2, 5kz0, 5l2s, 5l30, 5l3a, 5l4i, 5l4j, 5l4m, 5l7e, 5l7g, 5l7h, 5l8a, 5l9g, 5l9l, 5l9o, 5ld8, 5ldm, 5ldp, 5lli, 5lne, 5lom, 5lsg, 5lsh, 5lso, 5lud, 5lvl, 5lvq, 5lvr, 5lwm, 5lyn, 5lyr, 5lz4, 5lz5, 5lz7, 5m04, 5m17, 5m23, 5m25, 5m28, 5m5d, 5m77, 5m7s, 5m7u, 5mek, 5mes, 5mg2, 5mge, 5mgf, 5mgj, 5mgk, 5mkr, 5mks, 5mn1, 5mo8, 5mod, 5mpz, 5mqe, 5mrb, 5mrm, 5mro, 5mrp, 5mxf, 5my8, 5mz8, 5n0f, 5n17, 5n18, 5n1z, 5n6s, 5n93, 5n99, 5n9r, 5nbw, 5ndf, 5neb, 5ngz, 5nih, 5njz, 5nk2, 5nk3, 5nk4, 5nk6, 5nk7, 5nk8, 5nk9, 5nka, 5nkb, 5nkc, 5nkd, 5nkg, 5nkh, 5nki, 5nn5, 5nn6, 5nvv, 5nvw, 5nvx, 5nw0, 5nw1, 5nw2, 5nwi, 5o2d, 5o4f, 5o58, 5oei, 5oku, 5oot, 5op4, 5op5, 5oq8, 5orv, 5orw, 5os2, 5os4, 5os5, 5ose, 5ot8, 5ot9, 5ota, 5otc, 5ouh, 5ovr, 5ovx, 5sxm, 5sym, 5sz2, 5t19, 5t7s, 5t8o, 5t8p, 5t9u, 5t9w, 5t9z, 5ta2, 5ta4, 5tb6, 5tbe, 5tbm, 5tcj, 5tef, 5tp0, 5tpx, 5ttw, 5tuz, 5twj, 5u0w, 5u0y, 5u0z, 5u11, 5u12, 5u13, 5u14, 5u28, 5u49, 5u4b, 5u4d, 5u6j, 5u8c, 5ueu, 5uez, 5uf0, 5ufc, 5uff, 5ufp, 5ufr, 5ufs, 5uk8, 5ula, 5ult, 5uoo, 5uov, 5upe, 5upf, 5upz, 5ut6, 5uxf, 5v0n, 5v79, 5v7a, 5v82, 5vb5, 5vb6, 5vb7, 5vc3, 5vc4, 5vcv, 5vcw, 5vcy, 5vcz, 5vd0, 5vd1, 5vd2, 5vd3, 5vih, 5vij, 5vo1, 5voj, 5vp9, 5vsf, 5vsj, 5w1e, 5wa8, 5wa9, 5wal, 5wbm, 5wbo, 5wl0, 5wlo, 5wp5, 5wqc, 5wuk, 5wxh, 5wyx, 5wyz, 5x54, 5x62, 5x74, 5xg5, 5yjm, 6ayi, 6b4l, 6b4n, 6b4u, 6b7a, 6b7b, 6ep4, 6eqp, 6euw, 6eux, 6ezq
